# Generation and Analysis of Expressed Sequence Tags from *Olea europaea* L.

**DOI:** 10.1155/2010/757512

**Published:** 2010-12-08

**Authors:** Nehir Ozdemir Ozgenturk, Fatma Oruç, Ugur Sezerman, Alper Kuçukural, Senay Vural Korkut, Feriha Toksoz, Cemal Un

**Affiliations:** ^1^Department of Biology, Faculty of Science and Arts, Yildiz Technical University, Davutpasa Street 124, 34210 Merter/Istanbul, Turkey; ^2^Faculty of Engineering and Natural Sciences, Sabanci University, 34956 Tuzla/Istanbul, Turkey; ^3^Department of Biology, Faculty of Science, Ege University, 35100 Bornova/Izmir, Turkey

## Abstract

Olive (*Olea europaea* L.) is an important source of edible oil which was originated in Near-East region. In this study, two cDNA libraries were constructed from young olive leaves and immature olive fruits for generation of ESTs to discover the novel genes and search the function of unknown genes of olive. The randomly selected 3840 colonies were sequenced for EST collection from both libraries. Readable 2228 sequences for olive leaf and 1506 sequences for olive fruit were assembled into 205 and 69 contigs, respectively, whereas 2478 were singletons. Putative functions of all 2752 differentially expressed unique sequences were designated by gene homology based on BLAST and annotated using BLAST2GO. While 1339 ESTs show no homology to the database, 2024 ESTs have homology (under 80%) with hypothetical proteins, putative proteins, expressed proteins, and unknown proteins in NCBI-GenBank. 635 EST's unique genes sequence have been identified by over 80% homology to known function in other species which were not previously described in Olea family. Only 3.1% of total EST's was shown similarity with olive database existing in NCBI. This generated EST's data and consensus sequences were submitted to NCBI as valuable source for functional genome studies of olive.

## 1. Introduction

Oleacea family comprises 600 species in 24 genus and disseminates all around the world. The olive *Olea europaea *L, which is one of the first domesticated agricultural tree crops in the family *Oleaceae*, is cultivated mainly for both edible oil and table olives. The domestication of *Olea *europaea is supposed to be realized some 5700–5500 years ago in the Near-East [1]. Therefore, Anatolia is one of the most important areas of the olive origin of which over 86 varieties of Europea species are present in Turkey (Anatolia). It is known that olive is native to coastal areas of the Mediterranean region such as Spain, Italy, Greece, France, Turkey, Algeria, and Morocco. Olive is the most extensively cultivated fruit crop with its orchards cover about 9.8 mil. ha. in the world. According to the statistics published by FAO, Turkey is the fourth largest producer of olive oil in the world, after Spain, Italy, and Greece. Turkey is the first producer of black table olive in the world and Gemlik cuv. represents 80% of black table olives production in Turkey. Because of economical importance of Gemlik, a lot of research centers in Turkey continue their molecular and classical breeding program for this cultivar.

Most of the genetic studies in cultivated plants are focused on the understanding of genetic mechanisms and improvement of product quality and quantity. With the improvement of DNA-sequencing technology, large-scale single-pass cDNA sequencing is commonly used to obtain large expressed sequence tag (EST) collection which is generated with expressed gene at a particular stage and/or tissue of organism. The sequenced cDNA show direct information on the mature transcripts for coding part of the genome, so EST databases are very useful tools for gene and marker discovery, gene mapping, and functional studies. 

After the completion of the genome projects in different species, the number of ESTs has increased rapidly and become available in databases for further applications. Over 40 plant species EST libraries are currently available providing valuable resource for functional genomics studies [2–9]. 

 By using information from these EST databases the possible functions of many genes can be deduced by homologies to known genes. 

Although many molecular markers have been developed in olives [10–19], EST studies for olives are not sufficient. By the end of 2008 around one thousand ESTs were generated for searching development of olive fruits and deposited in NCBI database [20]. Before we submit the olive EST collection to database, there were just around 1126 sequences available in GenBank databases (February 2009). In this paper, we report a rich EST collection from two separate cDNA libraries constructed from the fresh germinated leaves and immature olive fruits for Turkish olive cultivar Gemlik. 2304 clones were sequenced from the leaf cDNA library and 1536 clones were sequenced from the fruit cDNA library. After removal of low-quality ESTs, generated 3734 high-quality olive ESTs were analyzed by using Phred-Phrap and Contig Assembly Program 3 (CAP3) software and were submitted to GenBank (dbEST). Annotation is performed by using BLAST and BLAST2GO.

## 2. Material and Method

The olive breeding line of *O.europea,* Gemlik cuv. (G 20/1) is used as a plant material research in this study. Plant materials were supplied by The Ataturk Central Horticultural Research Institute (ACHRI). 

### 2.1. Library Construction

Total RNA was isolated from 10 g fresh germinated leaves and immature olive fruits with the RNeasy Plant Miniprep kit (Qiagen) and pooled. mRNA was purified from total RNA using the Oligotex Spin-Column Protocol (Oligotex mRNA Mini Kit, Qiagen, Valencia, CA). The mRNAs were pooled and final concentration of mRNA was adjusted to 1–3 *μ*g. Two separate cDNA libraries were established with 1.5 *μ*g and 3 *μ*g mRNA leaf and immature olive fruit, respectively. cDNA libraries were constructed with the CloneMiner cDNA Library Construction Kit according to the manufacturer's instructions (Invitrogen, Carlsbad, CA, USA). Double-stranded cDNA was cloned into pDONR222 vector and transformed into E.coli strain DH5 (Invitrogen, Carlsbad, CA, USA). Each cDNA library was plated onto LB-kanamycin agar medium and individual grown clonies were picked into 384-well plates with SOB medium and inoculated overnight. After the addition of glycerol (10% v/v), the library was stored at −80°C.

### 2.2. Plasmid DNA Purification and DNA Sequencing

Plasmid DNA was isolated from randomly selected sixty clones with alkaline lysis method [21, 22]. Isolated DNA was digested with Bgl1701 and analyzed by a 1% agarose gel electrophoresis to identify insert size. 

Randomly selected 3840 clones were used as template for PCR amplification of the cloned cDNA by M13 universal primers. Automated sequencing was performed on an automated high-throughput pipeline using the ABI 3730 capillary sequencer (PE Applied Biosystems, Foster City, CA) at the Genome Sequencing Center, Washington University in St. Louis (WUSTL).

### 2.3. EST Analysis

EST sequences were trimmed of vector, adapter, and low-quality sequence by using Phred software [23, 24] (CodonCode Crop., Dedham, MA.) 106 low quality EST sequences were removed with the program Phred (version 3/19/99, default 20). The remaining 3734 EST sequences are reprocessed with “cross-match” application of Phrap for the vector sequence trimming [23, 24].

Total EST sequences, leaf, and fruit EST sequences, were assembled separately into contigs by using Contig Assembly Program 3 (Cap3) [25, 26]. The default values were used for all the parameters. Also, the assembly result was controlled with Consed/Autofinish software [27, 28]. Plausible functions for the established contigs were designated by gene homology based on BLAST. The biological meaning of the unique sequences was investigated according to gene ontology (GO) terms based on BLAST definitions using the program BLAST2GO which is a comprehensive bioinformatics tool for functional annotation and analysis of gene or protein sequences [29, 30].

## 3. Result

### 3.1. Quality of cDNA Libraries and Clustering of ESTs

Two separate, cDNA libraries were constructed from a pool of RNA extracted from young leaves and fruits independently. The insert size distribution ranged from 200 to 2500 bps in the leaf cDNA library which consisted of 2.4 × 10^6^ clones with an average insert length of 1.6 kb. In the immature olive fruit cDNA library, the average insert size was 1.1 kb (min 70 bp to max 1500 bp) and the library consisted of 2.2 × 10^5^ clones. After construction of cDNA libraries, 2304 clones were sequenced from the leaf library; 1536 clones were sequenced from the fruit library. Consequently, a total of 3840 EST sequences was generated. Raw EST sequence data was processed and base called by using Phred. The olive EST sequences were trimmed from the start and to the end of the sequences on the basis of trace quality to remove vector, adapter, and low-quality bases with the default value of 0.05. After this process, 106 clones were removed and the average length of 3734 ESTs was determined as 874 bp.

For contig assembly, designated 2228 high-quality leaf EST sequences and 1506 high-quality fruit EST sequences were analyzed as individual and total by program CAP3. While assembling the 2228 leaf EST sequences into 205 contigs, length ranged from 514 bases to 1924 bases, and the number of EST ranged from 2–33, 1506 fruit EST's were assembled in to 69 contig, length ranged from 461 bases to 1909 bases, and the number of EST ranged from 2–385 (Table 1). When we assembled two libraries together since there are some common genes expressed in the leaf as well as in the fruit, some of the ESTs obtained from the leaf and fruit established new contigs increasing the total contig number of the assmebled libraries to 299. Some of the singlets of the leaf and fruit libraries established new contigs when the libraries assembled together decreasing the total singlet number of the joint library by 100 to 2368. All 3734 EST sequences and the 249 of high-quality consensus sequences were submitted to GenBank (dbEST) and EST's can be accessed through the accession numbers GO242703–GO246436. Consensus sequences of olive can be reached on the accession numbers EZ421546–EZ421794.

### 3.2. Identification of ESTs' Putative Function

The annotation of the 3734 ESTs were designated by database search algorithms BLASTN for nucleic acids and the BLASTX for proteins at The National Center for Biotechnology Information (NCBI) web server. 

Among the 3734 ESTs, 682 of them (18.2%) showed significant sequence similarities to putative genes registrated in NCBI with score of ≥80 bits or e value ≤10^−10^ according to BLASTN similarity search against the nucleotid collection database (last verified on July 2010). The 1647 ESTs (44.1%) resulted in some hits but with weak similarity scores (≤80–40 bits) out of these 896 ESTs (23.9%) had a score between 60–79 bits and 751 ESTs (20.2%) had a score between 40–59 bits. The 1405 ESTs (37.7%), which gave very low similarity scores but stil gave some hits (0–39 hits) or gave no hits since they have no similarity to exisiting sequences in the databases, that is why they were classified in the “No hit” category. Some of the low scoring hits, may also be considered as no hits as well. But since the algorithms provided some hits we put them into weak similarity match category. BLASTN analysis against the nucleotid collection database between our EST and olea sequences in NCBl database has shown that there are only 116 ESTs have similarities, and 38% of these (45 ESTs) have 80% or higher homology (with the score of ≥80 bits). 96.9% of the ESTs generated by us in these studies are different than the ones in olive sequences database already presented by NCBI. On the other hand, with BLASTN analysis against EST database only 81 EST have similarities to olea ESTs in NCBI, and 29% of these have 80% or higher homology (with the score of ≥80 bits). 

According to the BLASTN result, 13 different total contigs sequences have similarities with *Olea Europaea *EST sequences in GenBank Table 2. These are: specifically those acting on the CH-OH group of donor with NAD+ or NADP+ as acceptor from oxidoreductases family “mannitol dehydrogenase1”, polypeptide that was employed the phases involved in photosystem II “photosystem II 10 kDa polypeptide mRNA”, “glycolate oxidase-like FMN-binding domain protein mRNA”, responsible for the shuttling of phospholipids and other fatty acid groups between cell membranes also able to bind acyl groups “plant lipid transfer protein mRNA”, most commonly known by the shorter name RuBisCO, is an enzyme that is used in the Calvin cycle to catalyze the first major step of carbon fixation, a process by which the atoms of atmospheric carbon dioxide are made available to organisms in the form of energy-rich molecules such as sucrose “ribulose-1,5-bisphosphate carboxylase/oxygenase activase mRNA”, enzyme that acts upon *β*1 − >4 bonds linking two glucose or glucose-substituted molecules “beta-glucosidase (bglc) mRNA”, vacuolar membrane protein in plants “tonoplast intrinsic protein (tip) mRNA”, to transmit signals between cells and binding large family of proteins “polyubiquitin OUB2 mRNA”, some sequences previously identified in olive and a protein that is involved in gluconeogenesis, the synthesis of glucose from smaller molecules “glyoxisomal malate dehydrogenase mRNA”. 

In addition to BLAST results, gene ontology (GO) annotations of the leaf, fruit and all contig sequences of *Olea Europea *L. cv. Gemlik were performed by using Blast2GO. The software performed BLASTX similarity search against the GenBank nonredundant protein database, retrieved GO terms for the top 20 BLAST results and annotated the sequences based on default criteria [29, 30]. GO terms were distributed among the biological process, molecular function and cellular component categories; see the following.

**Gene Ontology Results of Leaf, Fruit, and Total Contigs with the Program of BLAST2GO**

1. Leaf (Total 205 Contig)
  - Molecular function/number of contig (existent percentage):
    - protein binding/24 (11,7%)
    - ATP binding/13
    - DNA binding/9
    - Structural molecule activity/9
    - Iron ion binding/9
    - Peptidase activity/9
    - Nucleoside-triphosphatase activity/8
    - Carbon carbon lyase activity/7
    - Hydrolase activity, acting on ester bonds/7
    - GTP binding/7
    - Magnesium ion binding/7
    - Coenzyme binding/6
    - Transferase activity transfering acyl groups/6
    - Chlorophyl binding/6
    - Electron carrier activity/6
    - Zinc ion binding/6
    - Oxidoreductase activity acting on CH-OH 7group of donors/6
    - Transferase activity transfering phosphorus containing groups/6
    - Transmembrane transporter activity/6
    - Isomerase activity/5.
  - Cellular component/number of contig (existent percentage):
    - Integral to membrane/15
    - Photosystem II/15
    - Mitochondrion/14
    - Cytoplasmic membrane-bounded vesicle/8
    - Nucleus/8
    - Photosystem I/8
    - Chloroplast stroma/6
    - Cytosol/6
    - Chloroplast thylakoid membrane/6
    - Ribosome/6
    - Peroxisome/6.
  - Biological process/number of contig (existent percentage):
    - Transport/20 (9,7%)
    - Response to chemical stimulus/17
    - Response to stress/15
    - Nucleobase, nucleoside, nucleotide and nucleic asit metabolic proses/12
    - Glycolysis/11
    - Response to endogenous stimulus/11
    - Electron transport/11
    - Cellular lipid metabolic process/9
    - Translation/9
    - Regulation of cellular metabolic process/9
    - Photosynthesis, light harvesting/9
    - Organelle organization and biogenesis/8
    - Proteolysis/8
    - Amino acid biosynthetic process/7
    - Developmental process/7
    - Response to light stimulus/7
    - Protein-chromophore linkage/6
    - Monocarboxylic acid metabolic process/6.
2. Fruit (Total 69 Contig)
  - Molecular function/number of contig (existent percentage):
    - Hydrolase activity/9 (13%)
    - Transferase/8 (11,5%)
    - Metal ion binding/8 (11,5%)
    - Ion transmembrane transporter activity/6
    - Antiporter activity/6
    - Oxidoreductase activity/6
    - Cation binding/6
    - Nucleotide binding/6.
  - Cellular component/number of contig (existent percentage):
    - Mitochondrion/6
    - Integral to membrane/6
    - Vacuolar membrane/5
    - Chloroplast/4
    - Plastid/4
    - Membrane/3
    - Nucleus/2
    - Cytoplasm/2
    - Golgi aparatus oxygen evolving complex/1
    - Microtubulle/1
    - Cytosolic small ribosomal subunit/1.
  - Biological process/number of contig (existent percentage):
    - Cellular protein metabolic process/11 (15,4%)
    - Carboxylic acid metabolic process/10 (14,4%)
    - Response to stress/10(14,4%)
    - Biopolymer metabolic process/10 (14,4%)
    - Biosynthetic process/9 (13%)
    - Biological regulation/8 (11,5%)
    - Phosphorus metabolic process/7 (10.1%)
    - Nucleobase, nucleoside, nucleotide and nucleic asit metabolic proses/6
    - Ion transport/6
    - Cellular carbohydrate metabolic process/6
    - Rresponse to inorganic substance/6.
3. 3734 EST (Total 299 Contig)
  - Molecular function/number of contig (existent percentage):
    - ATP binding/19
    - DNA binding/11
    - Zinc ion binding/11
    - Iron ion binding/10
    - Structural constituent ribosome/9
    - Hydrolase activity, acting on ester bonds/9
    - Nucleoside-triphosphatase activity/9
    - Carbon carbon lyase activity/9
    - GTP binding/8
    - Carbon transmembrane transporter activity/8
    - Ligase activity/8
    - Calcium ion binding/8
    - Magnesium ion binding/8
    - Coenzyme binding/8
    - Isomerase activity/8
    - Kinase activity/7
    - Electron carrier activity/7
    - Chlorophyl binding/7
    - Antiporter activity/7
    - Endopeptidase activity/6
    - Oxidoreductase activity, acting on the aldehyde or oxo group of donors/6
    - Phosphotransferase activity, alchole groups as acceptor/6
    - Transferase activity transfering acyl groups/6
    - Unfolded protein binding/5
    - Oxidoreductase activity, acting on the CH-OH group of donors, NAD or NADP as acceptor/5.
  - Cellular component/number of contig (existent percentage):
    - Mitochondrion/23
    - Integral to membrane/22
    - Photosystem II/16
    - Cytoplasmic membrane-bounded vesicle/14
    - Nucleus/12
    - Ribosome/10
    - Photosystem I/9
    - Chloroplast stroma/7
    - Chloroplast thylakoid membrane/7
    - Cytosolic part/7
    - Endomembrane system/6
    - Cytoskeleton/6
    - Vacuolar membrane/6
    - Peroxisome/6.
  - Biological process/number of contig (existent percentage)
    - Translation/14
    - Electron transport/13
    - Glycolysis/12
    - Organelle organization and biogenesis/12
    - Response to endogenous stimulus/11
    - Cellular lipid metabolic process/11
    - Photosynthesis, light harvesting/10
    - Proteolysis/10
    - Protein folding/9
    - Response to salt stress/8
    - Coenzyme metabolic process/8
    - Lipid biosynthetic process/7
    - Phosphorylation/7
    - Response to cold stress/7
    - Response to light stimulus/7
    - Developmental process/7
    - Protein-chromophore linkage/7
    - Amino acid biosynthetic process/7
    - Reductive pentose-phosphate cycle/7
    - Monocarboxylic acid metabolic process/6
    - Biopolymer biosynthetic process/6
    - Response to oxidative stress/6
    - Protein catabolic process/6
    - Pesponse to metal ion/6
    - Cellular di-,tri-valent inorganic cation homeostasis/6
    - Metal ion transport/6
    - RNA metabolic process/6
    - Secondary metabolic process/6
    - Regulation of transcription/5
    - Establishment of cellular localization/5.

20 different types of molecular functions were found for 162 leaf contigs by Blast2GO program. Also, Blast2GO results showed that 47 fruit contigs have 8 different molecular function as GO terms, and the contigs that were prepared from all ESTs have 25 different types of molecular functions in 205 contigs. The common molecular function GO terms for all three results are “hydrolase activity”, “transferase activity”, “transmembrane transporter activity”, “oxidoreductase activity,” and “ion binding”. Most of the assigned functional class (11,7%) is binding proteins for the sequences obtained from the leaves. Fruit contigs also have binding proteins as functional class but not as common as leaf contigs. All molecular function results from revealed BLAST2GO program are shown previously in the paper. 

**Figure 1:**
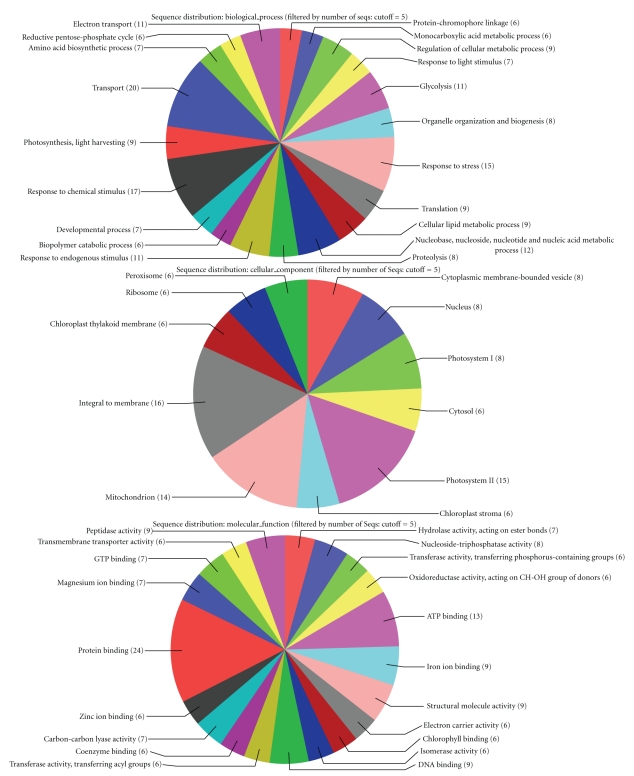
GO terms distribution in the biological process show with circle graphs for leaf (a), fruit (b), and total contigs (c).

 The biological process category refers to a biological objective to which a gene contributes, but does not identify pathways. Biological process results are identified by BLAST2GO program like molecular function results. Results are similar for all three contig groups. Especially “carboxylic acid metabolic process”, “biosynthetic process”, “response to stress”, “transport”, “biopolymer metabolic process”, and “nucleobase, nucleoside, nucleotide and nucleic asit metabolic process” are common for all three results. But there were a lot of different GO terms for biological process results. For instance, in fruit contigs “phosphorus metabolic process”, “biological regulation”, “cellular carbohydrate metabolic process”, “cellular protein metabolic process” and “response to inorganic substance” GO terms were not seen in leaf contigs. Some of GO terms like “response to chemical stimulus”, “response to endogenous stimulus”, “cellular lipid metabolic process”, “glycolysis”, “proteolysis” and “protein-chromophore linkage” were not seen in fruit contigs. All the observed differences and similarities between contig groups are summarized before in the paper. When in Figure 1 the biological process which is most observed for leaf in GO terms are transport, response to chemical stimulus, response to stress, in total contigs, GO terms of translation, electron transport, glycolysis, and in fruit, cellular protein metabolic process, carboxylic acid metabolic process, and response to stress are the most observed ones. Facing different GO terms in total contigs depends on the fact that the different sequences among the leaf and fruit contigs do form new consensus sequences.

The final GO term category identifies the locations in the cell where the gene products are found. The *Olea europaea* gene products were found generally associated with the cellular components, in the intracellular space or in organelles such as the mitochondrion, cytoskeleton, vacuolar membrane, peroxisome, and ribosome. Despite the fact that the most represented GO terms for cellular components of all contigs are integral to membrane and mitochondrion, in the meantime, as expected photosystem II has also been most observed GO term for the leaf.

## 4. Discussion

The EST's give very remarkable information about gene expression patterns at a certain stage of the organism. ESTs have been used for gene discovery [31, 32] tissue- or stage-specific gene expression [33] and alternative splicing [34]. In this project, we aimed to obtain more information about olive genome, and we have planned to produce a large EST collection for *Olea Europea *L. which has limited number of ESTs in databases. In order to achieve this goal of creating a larger and richer collection, we have constructed two different cDNA libraries from leaves and fruits for increasing our chance to capture different genes. 

According to BLASTN result, we have observed some common putative genes between leaves and fruit contigs assembled by CAP3 such as reductase, cytochrome P450, GDP-mannose-3′,5′-epimerase (GME), tubulin, ascorbate peroxidase, beta-glucosidase, polyubiquitin, aldolase-like protein, ubiquitin, and chlorophyll a/b binding protein. Among the assembled leaves contigs some specific putative genes were observed such as asparagine synthetase (AS), germacrene D synthase, desacetoxyvindoline 4-hydroxylase-like (D4H), plastid transketolase 1, ABC transporter family protein, glutamate synthase 1, chloroplast ferredoxin I, glyceraldehyde-3-phosphate dehydrogenase, chlorophyll a/b-binding protein, malate dehydrogenase, alcohol dehydrogenase, and mannitol dehydrogenase 1. Equally among the assembled fruit contigs have some different putative genes than leaves such as SDH2-1, UDP-glucuronate decarboxylase 3, cytoplasmic ribosomal protein, aspartic protease, S-RNase-binding protein, chloroplast oxygen-evolving protein, elongation factor 1 alpha subunit, myb-related transcription factor, Tic20-like protein, and Ca2+ antiporter/cation exchanger. Since less than 10% of olive genes were tagged in each tissue, in this study, some of the GO terms occurring on one tissue and not on the other tissue could be due to the less representative ESTs obtained or sampling variation and may not infer to tissue-specific genes. 

On the other hand, the Blast2GO analysis of assembled EST's enabled the identification of GO terms on three different categories, such as molecular function, biological process, and cellular location. While the leaf contigs gave hits on 20 different functional classes and fruit contigs gave hits on 8 functional classes, but contigs obtained from the combined library yielded in hits on 25 functional classes, some of them were not observed in functional classes obtained from the leaf and fruit libraries alone. This may be the result of new contigs generated by the combination of the libraries which are giving hits to genes belonging to new functional classes which maybe expressed both in the leaf and the fruit tissues.

It has been the widest olive genome EST collection of *Olea Europea *L. cv. Gemlik which was constructed to the date. The number of ESTs of *Olea europea* is 4860 in NCBI (last verified on May 2010), and 3734 out of this figure were generated within this study. This project has dramatically increased the number of Olive ESTs in NCBI GenBank database which is a very useful source for the scientists working on olive genome or on comparative genome researches. For further researches, more ESTs should be generated and be annotated in order to increase the identified number of expressed olive genes for functional analysis.

## Figures and Tables

**Table 1:** The assembly analysis of EST for two cDNA libraries independently and together by CAP3.

	Leaf	Fruit	Total
Number of ESTs	2228	1506	3734
Number of contig	205	69	299
Number of singlet	1.591	887	2.368
Average length of contigs	2194 bp	1912 bp	2134 bp
Number of EST range in the contig	2–33	2–385	2–379

**Table 2:** Homolog genes with *Olea Europaea *consensus EST sequences in GenBank.

Contig name	Homology of *Olea europea *in NCBI data base	Query coverage (%)	Max. Ident. (%)	Length of Contig (bp)	The number of EST in the contig
Contig 7	*Olea europaea* putative mannitol dehydrogenase 1 (MTD1) mRNA	56	86	895	2
Contig 14	*Olea europaea* photosystem II 10 kDa polypeptide mRNA, partial cds	30	99	724	4
Contig 24	*Olea europaea* putative glycolate oxidase-like FMN-binding domain protein mRNA	22	99	2819	9
Contig 85	*Olea europaea* putative plant lipid transfer protein mRNA	25	100	914	5
Contig 93	*Olea europaea* Cu/Zn superoxide dismutase (ole e 5 allergen)	79	93	891	2
Contig 98	*Olea europaea* putative cytochrome P450 mRNA, partial cds	28	99	1756	8
Contig 111	*Olea europaea* putative ribulose-1,5-bisphosphate carboxylase/oxygenase activase mRNA, partial cds	43	98	1797	22
Contig 137	*Olea europaea* subsp. europaea beta-glucosidase (bglc) mRNA, complete cds	93	98	2018	13
Contig 155	*Olea europaea* tonoplast intrinsic protein (tip) mRNA, complete cds	87	86	911	2
Contig 157	*Olea europaea* polyubiquitin OUB2 mRNA, complete cds	89	91	1393	7
Contig 169	*Olea europaea* cultivar Bianchera tRNA-His (trnH) gene, partial sequence; trnH-psbA intergenic spacer, complete sequence; PSII 32 kDa protein (psbA) gene, complete cds; psbA-trnK intergenic spacer, complete sequence; and tRNA-Lys (trnK) gene, partial sequence; chloroplast	97	96	1225	2
Contig 201	*Olea europaea* putative glyoxisomal malate dehydrogenase mRNA, partial cds	46	97	1383	2
Contig 255	*Olea europaea* putative metallophosphatase/diphosphonucleotide phosphatase 1 mRNA, partial cds	28	96	969	2

## References

[1] Zohary M, Hopf M (1994). *Domestication of Plants in the Old World*.

[2] Martienssen RA (2000). Weeding out the genes: the *Arabidopsis* genome project. *Functional and Integrative Genomics*.

[3] Yamamoto K, Sasaki T (1997). Large-scale EST sequencing in rice. *Plant Molecular Biology*.

[4] Yu J, Hu S, Wang J (2002). A draft sequence of the rice genome (*Oryza sativa* L. ssp. *indica*). *Science*.

[5] Van der Hoeven R, Ronning C, Giovannoni J, Martin G, Tanksley S (2002). Deductions about the number, organization, and evolution of genes in the tomato genome based on analysis of a large expressed sequence tag collection and selective genomic sequencing. *Plant Cell*.

[6] Moyle R, Fairbairn DJ, Ripi J, Crowe M, Botella JR (2005). Developing pineapple fruit has a small transcriptome dominated by metallothionein. *Journal of Experimental Botany*.

[7] Moser C, Segala C, Fontana P (2005). Comparative analysis of expressed sequence tags from different organs of Vitis vinifera L. *Functional and Integrative Genomics*.

[8] Grimplet J, Romieu C, Audergon J-M (2005). Transcriptomic study of apricot fruit (Prunus armeniaca) ripening among 13 006 expressed sequence tags. *Physiologia Plantarum*.

[9] Newcomb RD, Crowhurst RN, Gleave AP (2006). Analyses of expressed sequence tags from apple. *Plant Physiology*.

[10] Wiesman Z, Avidan N, Lavee S, Quebedeaux B (1998). Molecular characterization of common olive varieties in Israel and the West Bank using randomly amplified polymorphic DNA (RAPD) markers. *Journal of the American Society for Horticultural Science*.

[11] Mekuria GT, Collins GG, Sedgley M (1999). Genetic variability between different accessions of some common commercial olive cultivars. *Journal of Horticultural Science and Biotechnology*.

[12] Angiolillo A, Mencuccini M, Baldoni L (1999). Olive genetic diversity assessed using amplified fragment length polymorphisms. *Theoretical and Applied Genetics*.

[13] Besnard G, Green PS, Bervillé A (2002). The genus *Olea*: molecular approaches of its structure and relationships to other *Oleaceae*. *Acta Botanica Gallica*.

[14] Rallo P, Dorado G, Martín A (2000). Development of simple sequence repeats (SSRs) in olive tree (Olea europaea L.). *Theoretical and Applied Genetics*.

[15] Belaj A, Trujillo I, De la Rosa R, Rallo L, Giménez MJ (2001). Polymorphism and discrimination capacity of randomly amplified polymorphic markers in an olive germplasm bank. *Journal of the American Society for Horticultural Science*.

[16] Besnard G, Bervillé A (2002). On chloroplast DNA variations in the olive (*Olea europaea* L.) complex: comparison of RFLP and PCR polymorphisms. *Theoretical and Applied Genetics*.

[17] de Caraffa VB, Maury J, Gambotti C, Breton C, Bervillé A, Giannettini J (2002). Mitochondrial DNA variation and RAPD mark oleasters, olive and feral olive from Western and Eastern Mediterranean. *Theoretical and Applied Genetics*.

[18] Cipriani G, Marrazzo MT, Marconi R, Cimato A, Testolin R (2002). Microsatellite markers isolated in olive (*Olea europaea* L.) are suitable for individual fingerprinting and reveal polymorphism within ancient cultivars. *Theoretical and Applied Genetics*.

[19] Sefc KM, Lopes MS, Mendonça D, Dos Santos MR, Machado LMdaC, Machado ADaC (2000). Identification of microsatellite loci in olive (*Olea europaea*) and their characterization in Italian and Iberian olive trees. *Molecular Ecology*.

[20] Galla G, Barcaccia G, Ramina A (2009). Computational annotation og genes differentially expressed along olive fruit development. *BMC Plant Biology*.

[21] Sambrook J, Fritsch EF, Maniatis T (1989). *Molecular Cloning: A Laboratory Manual*.

[22] Feliciello I, Chinali G (1993). A modified alkaline lysis method for the preparation of highly purified plasmid DNA from Escherichia coli. *Analytical Biochemistry*.

[23] Ewing B, Green P (1998). Base-calling of automated sequencer traces using phred. II. Error probabilities. *Genome Research*.

[24] Ewing B, Hillier L, Wendl MC, Green P (1998). Base-calling of automated sequencer traces using phred. I. Accuracy assessment. *Genome Research*.

[25] Huang X (1992). A contig assembly program based on sensitive detection of fragment overlaps. *Genomics*.

[26] Huang X, Madan A (1999). CAP3: a DNA sequence assembly program. *Genome Research*.

[27] Gordon D, Abajian C, Green P (1998). Consed: a graphical tool for sequence finishing. *Genome Research*.

[28] Gordon D, Desmarais C, Green P (2001). Automated finishing with autofinish. *Genome Research*.

[29] Conesa A, Götz S, García-Gómez JM, Terol J, Talón M, Robles M (2005). Blast2GO: a universal tool for annotation, visualization and analysis in functional genomics research. *Bioinformatics*.

[30] Conesa A, Götz S (2008). Blast2GO: a comprehensive suite for functional analysis in plant genomics. *International Journal of Plant Genomics*.

[31] Schmitt AO, Specht T, Beckmann G (1999). Exhaustive mining of EST libraries for genes differentially expressed in normal and tumour tissues. *Nucleic Acids Research*.

[32] Lee Y, Tsai J, Sunkara S (2005). The TIGR Gene Indices: clustering and assembling EST and know genes and integration with eukaryotic genomes. *Nucleic Acids Research*.

[33] Audic S, Claverie J-M (1997). The significance of digital gene expression profiles. *Genome Research*.

[34] Gupta S, Zink D, Korn B, Vingron M, Haas SA (2004). Genome wide identification and classification of alternative splicing based on EST data. *Bioinformatics*.

